# A comparative study of two PODXL antibodies in 840 colorectal cancer patients

**DOI:** 10.1186/1471-2407-14-494

**Published:** 2014-07-08

**Authors:** Tuomas Kaprio, Jaana Hagström, Christian Fermér, Harri Mustonen, Camilla Böckelman, Olle Nilsson, Caj Haglund

**Affiliations:** 1 Department of Surgery, Helsinki University Central Hospital, P.O. Box 440, 00029 HUS Helsinki, Finland; 2 Research Programs Unit, Translational Cancer Biology, University of Helsinki, Helsinki, Finland; 3 Fujirebio Diagnostics AB, Elof Lindälvs gata 13, SE-414 58 Göteborg, Sweden; 4 Onson Consulting, Södra vägen 2, SE-412 54 Göteborg, Sweden; 5 Department of Pathology, Haartman Institute, University of Helsinki and HUSLAB, Helsinki FIN-00014 HY, Finland; 6 Department of Surgery, Vaasa Central Hospital, Sandviksgatan 2-4, 65100 Vaasa VASA, Finland

**Keywords:** Colorectal cancer, Podocalyxin, Prognosis

## Abstract

**Background:**

Podocalyxin (PODXL) is a transmembrane sialomucin, whose aberrant expression and/or allelic variation associates with poor prognosis and unfavourable clinicopathological characteristics in different cancers. Membranous expression of PODXL has been suggested to be an independent marker of poor prognosis in colorectal cancer (CRC), and previously by an in-house monoclonal antibody, we showed that also cytoplasmic overexpression of PODXL predicts poor prognosis. The aim of this study was to compare two PODXL antibodies with different epitopes case-by-case in CRC patients.

**Methods:**

Of 840 consecutively operated CRC patients from Helsinki University Central Hospital, PODXL expression by polyclonal HPA 2110 antibody was evaluated from 780. Associations of PODXL expression with clinicopathological parameters and the impact of PODXL expression on survival were assessed. Kappa-value was used to assess the comparability of the two antibodies.

**Results:**

Membranous PODXL expression associated with unfavourable clinicopathological parameters and with higher risk for disease-specific death from CRC within 5 years (unadjusted hazard ratio (HR) = 1.90; 95% confidence interval (CI) (1.32-2.75); adjusted HR = 1.64; 95% CI (1.11-2.43)). The comparability of expressions by the two antibodies was low (kappa =0.219, standard error 0.060, p < 0.0001). Combination of two antibodies identified a group of patients with even worse prognosis (unadjusted HR = 6.00; 95% CI (3.27-13.0); adjusted HR = 2.14; 95% CI (1.12-4.07)).

**Conclusion:**

Membranous expression by the polyclonal PODXL antibody and cytoplasmic overexpression by the monocolonal PODXL antibody are both independent markers of poor prognosis, but they recognise different groups of patients, both of which have poor prognosis. The combined use of the antibodies reveals a group with an even worse prognosis. The biological reasons for the difference between antibodies warrant further studies.

## Background

The incidence of colorectal cancer (CRC) is increasing, especially in the Western world; more than one million new cases are diagnosed yearly. Even in good series the survival is about 60%, disease stage at diagnosis being the most important prognostic factor. To be able to more precisely predict outcome of patients we need prognostic factors in addition to clinicopathological stage [1].

In most countries stage III patients are routinely treated with adjuvant therapy, which gives a 10% absolute increase in 5-year survival. The advantage of adjuvant therapy in stage II patients is not that clear. It would be important to identify those stage II patients who benefit from postoperative treatment [2].

Podocalyxin-like 1 (PODXL) was originally found in kidney podocytes [3], but it is also expressed by vascular [4] and breast epithelium [5], and haematopoietic progenitors [6]. It is an anti-adhesive transmembrane glycoprotein that can be comprehensively sialyted and O-glycosylated. Estimated peptide mass for PODXL is 59 kDA, and prostranslational processing yields a mature glykoprotein of 165 kDA [7]. PODXL is recognised as a stem cell marker [8], closely related to CD34 and endoglycan. It regulates cell morphology and adhesion through its connections to intracellular proteins and to extracellular ligands [9-12]. The role of PODXL in cancer is not fully understood, but it seems to participate in epithelial-mesenchymal transition [13], and it interacts with different mediators of metastasis [10-12,14,15].

In many cancers, such as renal cell carcinoma, breast, colorectal, urothelial bladder, testicular, and pancreatic cancer PODXL has been reported to be expressed aberrantly and in the first four also to be an independent marker of poor prognosis [5,10,16-19]. Membranous PODXL expression has been suggested to correlate with poor prognosis in CRC and urothelial bladder cancer [17,18,20]. Germline variants of PODXL was associated with the development of prostate cancer and also with the presence of a more aggressive form [14]. The presence of missense mutations increased the risk for development of cancer by 50% and an in-frame deletion was linked to more aggressive tumours [14]. We recently showed by using a novel monoclonal antibody (mAb) that high cytoplasmic expression of PODXL is a marker of poor prognosis in CRC [21].

Because of apparent difference in PODXL expression depending on antibodies used we decided to compare PODXL expression, by our own in-house HES9 mAb and by a commercially available polyclonal antibody (pAb) used in other studies [17,18], case-by-case in a cohort of 840 CRC patients.

## Methods

### Patients

The study population comprised 840 consecutive colorectal cancer patients operated in 1983–2001 at the Department of Surgery, Helsinki University Central Hospital. The Finnish Population Register Centre provided follow-up vital status data needed to compute survival statistics, and Statistics Finland provided cause of death for all those deceased. Median age at diagnosis was 66, with a median follow-up of 5.1 years (range 0–25.8). The 5-year disease-specific survival rate was 58.9% (95% Cl 55.0-62.8%). This study was approved by the Surgical Ethics Committee of Helsinki University Central Hospital (Dnro HUS 226/E6/06, extension TMK02 §66 17.4.2013) and the National Supervisory Authority of Welfare and Health (Valvira Dnro 10041/06.01.03.01/2012).

### Preparation of tumour tissue microarrays

Formalin-fixed and paraffin-embedded tumour samples came from the archives of Department of Pathology, Helsinki University Central Hospital. Representative areas of tumour samples on haematoxylin- and eosin-stained tumour slides were marked by an experienced pathologist. Three 1.0-mm-diameter punches taken from each sample were mounted on recipient paraffin block with a semiautomatic tissue microarray instrument (TMA) (Beecher Instruments, Silver Spring, MD, USA) as described [22].

### Antibodies

The monoclonal in-house antibody (HES9) recognises amino acid residues 189–192 of PODXL. The polyclonal antibody (HPA 2110, Atlas Antibodies, Stockholm, Sweden) recognises amino acid residues 278–415 of PODXL. Both epitopes are in the extracellular part of PODXL. Of four protein coding PODXL splice variants, the epitope sequence of the pAb matches three with 100% (PODXL 001, 005, and 201, The Human Protein Atlas). The fourth splice variant matches with 87% (PODXL 202). The epitope sequence of the mAb HES9 matches all splice variants with 100%. The antibodies have been described in detail [21,23,24].

### Immunohistochemistry

TMA-blocks were freshly cut into 4-μm sections. After deparaffinization in xylene and rehydration through a gradually decreasing concentration of ethanol to distilled water, slides were treated in a PreTreatment module (Lab Vision Corp., Fremont, CA, USA) in Tris–HCl (pH 8.5) buffer for 20 minutes at 98°C for antigen retrieval. For the staining procedure by the Dako REAL EnVision Detection system, Peroxidase/DAB+, Rabbit/Mouse (Dako, Glostrup, Denmark) an Autostainer 480 (Lab Vision) was used. Tissues were incubated with the mAb (dilution 1:500 = 5 μg/ml) or pAb (dilution 1:250) for one hour at room temperature. In every staining series renal tissue served as positive control.

### Scoring of samples

As reported PODXL expression by the HES9 mAb was cytoplasmic and often granular. Positivity in tumour cells was uniform, with no nuclear expression [21]. By the pAb PODXL expression was cytoplasmic, with no nuclear expression. In some cases there were a distinct membranous positivity, even with weak cytoplasmic positivity. For the mAb negative cytoplasmic staining was scored as 0, weakly positive as 1, moderately positive as 2, and strongly positive as 3. For the pAb cytoplasmic staining was scored 0–2 (negative-moderate-strong) and in case of distinct membranous staining as 3, regardless of the intensity of the cytoplasmic staining [17]. Stainings were scored independently by T.K. and J.H., who were blinded to clinical data and outcome. Differences in scoring were discussed until consensus.

### Statistical analyses

For statistical purposes, categories of PODXL expression were dichotomised into low (0–2) and high (3) for the mAb and into non-membranous (0–2) and membranous (3) for the pAb. To study the two antibodies together a categorization with three classes was created; low (mAb: low and pAb: non-membranous), moderate (either mAb: high or pAb: membranous), and high (mAb: high and pAb: membranous). The antibodies were also categorized as weak (mAb: low and pAb: non-membranous) and strong (mAb: high and/or pAb: membranous). Evaluation of the association between PODXL expression and clinicopathological parameters was done by the Fisher exact-test or the linear-by-linear association test for ordered parameters. Kappa-value was used for testing the concordance of PODXL expression according to mAb and pAb. Disease-specific overall survival was counted from date of surgery to date of death from colorectal cancer, or until end of follow-up. Survival analysis was done by the Kaplan-Meier method and compared by the log rank test. The Cox regression proportional hazard model served for uni- and multivariable survival analysis, adjusted for sex, age, Dukes classification, and differentiation. Testing of the Cox model assumption of constant hazard ratios over time involved the inclusion of a time-dependent covariate separately for each testable variable. Hazard ratios of differentiation and Dukes class D were analyzed in two periods (0 to 1.25 and 1.25 to 5 years) in order to meet the assumptions of the Cox model, and the time-dependent Cox model was used. Interaction terms were considered, but no significant interactions were found. All tests were two-sided. A p-value of 0.05 was considered significant. All statistical analyses were done with SPSS version 20.0 (IBM SPSS Statistics, version 20.0 for Mac; SPSS, Inc., Chicago, IL, USA, an IBM Company).

## Results

### Immunohistochemical staining by the polyclonal antibody

PODXL expression by the pAb was cytoplasmic in tumour cells, but in some cases a distinct membranous expression was seen, which did not always correlate with intensity of cytoplasmic expression. Such distinct membranous staining was not seen with the mAb HES9 [21].

**Figure 1:**
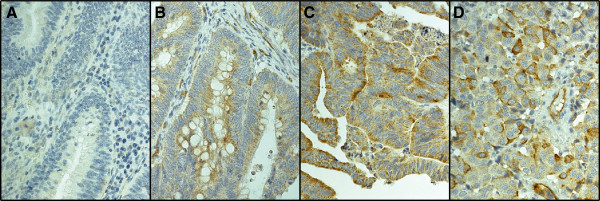
**Immunohistochemical staining pattern of PODXL by polyclonal antibody HPA 2110.** Representative images of PODXL expression in colorectal cancer. **(A)** PODXL-negative, **(B)** moderate cytoplasmic positivity, **(C)** strong cytoplasmic positivity, and **(D)** positive membranous immunoreactivity. Original magnification was × 40.

**Figure 2:**
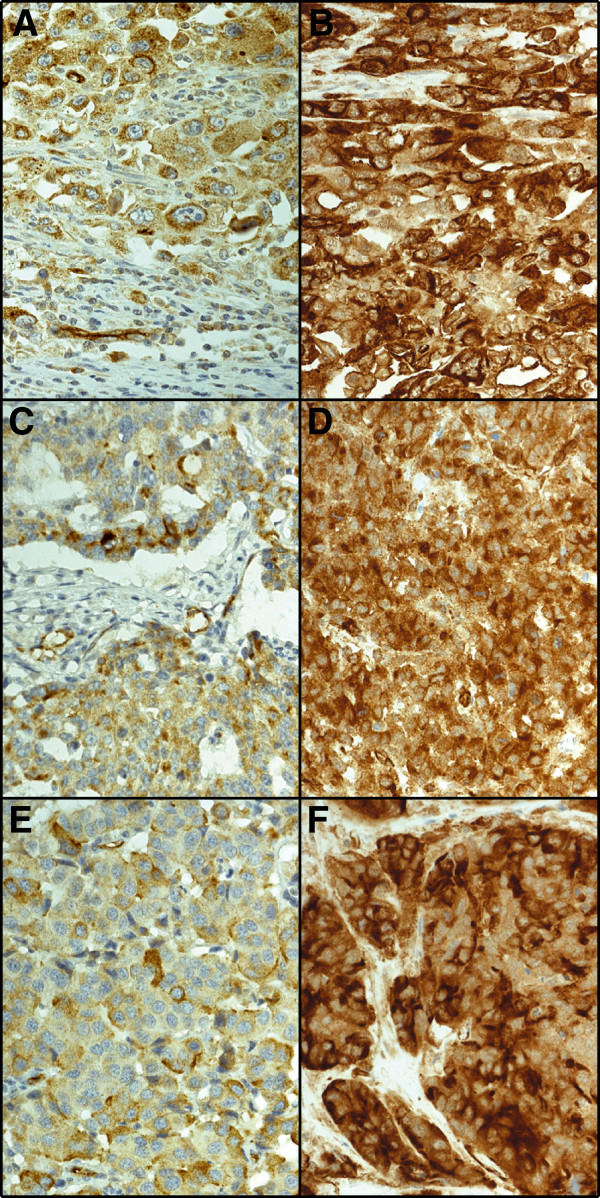
**Case-by-case comparison of immunohistochemical stainings by monoclonal antibody HES9 and polyclonal antibody HPA 2110 in colorectal cance.** Comparative images of three colorectal cancer tumour samples with membranous staining pattern by the polyclonal antibody **(A, C, E)** or strong cytoplasmic positivity by the monoclonal antibody **(B, D, F)**.

Of 840 tumours represented in the TMA, PODXL staining with pAb could be evaluated in 780 (92.6%); 46 (5.9%) had no cytoplasmic positivity, 322 (41.2%) showed moderate cytoplasmic staining, 349 (44.7%) strong cytoplasmic staining, and 63 (8.1%) positive staining with distinctive membranous staining. Representative images of pAb stainings are shown in Figure 1. Comparative images of high cytoplasmic staining by the mAb and membranous staining by the pAb in same tumours are shown in Figure 2. The staining results by the mAb HES9 have been described previously [21].

### Association of PODXL expression with clinicopathological parameters

There was a strong association between membranous PODXL expression and poor differentiation (p < 0.0001) and advanced stage (p < 0.001). Membranous PODXL expression did not associate with age, gender, tumour location (right vs left hemicolon or colon vs rectum), or tumour histology (Table 1). The corresponding results for the mAb HES9 have been described [21].

**Table 1:** Association of PODXL expression and clinicopathological parameters by polyclonal antibody HPA 2110

	**PODXL expression**		
	**Non-membranous**	**Membranous**	
**n (%)**	**717 (91.9)**	**63 (8.1)**	**p-value**
**Age, years**			
<65	299 (41.7)	32 (50.8)	0.184
≥ 65	418 (48.3)	31 (49.2)	
**Gender**			
Male	398 (55.5)	31 (49.2)	0.357
Female	319 (44.5)	32 (50.8)	
**Dukes**			
A	111 (15.5)	2 (3.2)	< 0.001
B	258 (36.0)	17 (27.0)	
C	191 (26.6)	22 (34.9)	
D	157 (21.9)	22 (34.9)	
**Grade (WHO)**			
1	27 (3.8)	0 (0)	< 0.0001
2	511 (71.8)	27 (42.9)	
3	155 (21.8)	30 (47.6)	
4	19 (2.7)	6 (9.5)	
Missing	5		
**Location**			
Colon	370 (51.6)	35 (55.6)	0.548
Rectum	347 (48.4)	28 (44.4)	
**Side**			
Right	193 (26.9)	21 (33.3)	0.274
Left	542 (73.1)	42 (66.7)	
**Histology**			
Adenomatous	644 (89.9)	57 (90.5)	1.000
Mucinous	72 (10.1)	6 (9.5)	
Missing	1		

Fisher exact-test was used for 2 × 2 tables and linear-by-linear association test for tables with more than two rows. Missing data is not included in the analyses.

### Comparison of PODXL expression by mono- and polyclonal antibodies

The agreement of expression of the two antibodies across cases was low (kappa-value = 0.219, standard error 0.060, p < 0.0001) using dichotomous values for both antibodies. Of the distinctive strong staining by mAb (n = 44) and membranous staining by pAb (n = 63) only 14 tumours were shared.

### Association of PODXL expression with clinicopathological parameters by monoclonal and polyclonal antibodies combined

Analysis of combined PODXL expression (low-moderate-high categories) with clinicopathological parameters showed significant associations between high PODXL expression and poor differentiation (p < 0.0001), advanced stage (p < 0.0001), and tumour side (p = 0.003). High expression did not associate with age, gender, tumour location (colon vs rectum), nor with tumour histology (Table 2). Association of the clinicopathological parameters with the strong PODXL expression (weak-strong categories) were similar (data not shown).

**Table 2:** Association of clinicopathological parameters and PODXL expression by polyclonal and monoclonal antibodies combined

	**PODXL expression**			
	**Low**	**Moderate**	**High**	
**n (%)**	**714 (88.4)**	**79 (9.8)**	**14 (1.7)**	**p-value**
**Age, years**				
<65	300 (42.0)	38 (48.1)	5 (35.7)	0.643
≥ 65	414 (58.0)	41 (51.9)	9 (64.3)	
**Gender**				
Male	398 (55.7)	41 (51.9)	7 (50.0)	0.450
Female	316 (44.3)	38 (48.1)	7 (50.0)	
**Dukes**				
A	113 (15.8)	4 (5.1)	0 (0.0)	< 0.0001
B	257 (36.0)	26 (32.9)	2 (14.3)	
C	187 (26.2)	32 (40.5)	2 (14.3)	
D	157 (22.0)	17 (21.5)	10 (71.4)	
**Grade (WHO)**				
1	28 (3.9)	0 (0.0)	0	< 0.0001
2	513 (72.4)	32 (40.5)	3 (21.4)	
3	149 (21.0)	37 (46.8)	9 (64.3)	
4	19 (2.7)	10 (12.7)	2 (14.3)	
Missing	5			
**Location**				
Colon	362 (50.7)	49 (62.0)	7 (50.0)	0.168
Rectum	352 (49.3)	30 (38.0)	7 (50.0)	
**Side**				
Right	185 (25.9)	32 (40.5)	6 (42.9)	0.003
Left	529 (74.1)	47 (59.5)	8 (57.1)	
**Histology**				
Adenomatous	639 (89.6)	70 (88.6)	13 (92.9)	0.879
Mucinous	74 (10.4)	9 (11.4)	1 (7.1)	
Missing	1			

Linear-by-linear association test was used for this table. Missing data is not included in the analyses.

### Survival analysis

**Figure 3:**
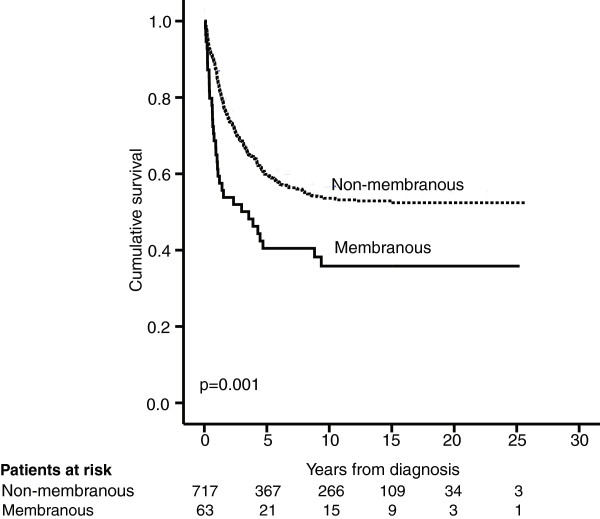
**Membranous PODXL expression by polyclonal antibody HPA 2110 is a marker of poor prognosis in colorectal cancer.** Disease-specific survival analysis according to the Kaplan-Meier method for membranous PODXL expression by the polyclonal antibody HPA 2110 in colorectal cancer. Log-rank test was used here.

**Figure 4:**
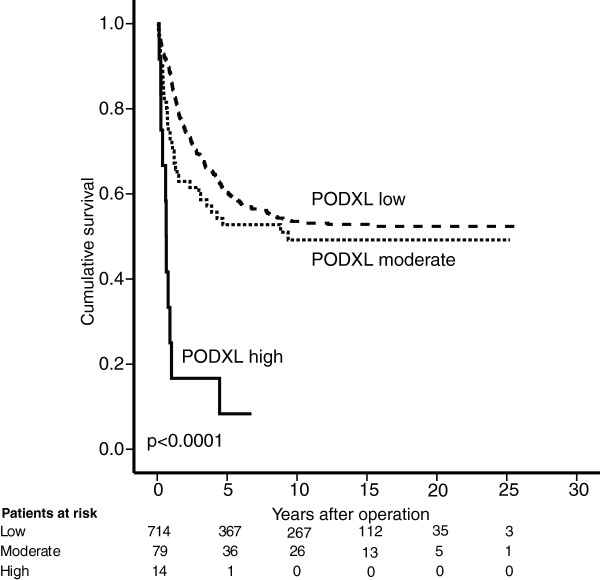
**Concomitant positivity by two PODXL antibodies identifies a group with very poor prognosis.** Disease-specific survival analysis according to the Kaplan-Meier method for combined expression of PODXL by polyclonal antibody HPA 2110 and monoclonal antibody HES9. Concomitant membranous positivity by the polyclonal antibody and high cytoplasmic positivity by the monoclonal antibody identifies a group with an even worse prognosis in colorectal cancer. Global log-rank was the test used here.

For colorectal cancer patients with membranous PODXL expression by polyclonal antibody disease-specific survival (DSS) was significantly poorer (p = 0.001). Five-year DSS was 40.5% (95% CI 27.4-53.6%) for patients with membranous PODXL expression compared to 60.0% (95% CI 56.3-63.7%) for non-membranous expression (Figure 3). Results were similar for the combination of pAb and mAb (weak-strong), but the group with poor prognosis was larger (n = 93 vs 63) (data not shown).Combination (low-moderate-high) of mAb and pAb showed a significantly poorer DSS for colorectal cancer patients with high expression compared to low expression (p < 0.0001) or moderate expression (p < 0.0001). No statistically significant difference in DSS was seen between those with low and moderate expression (p = 0.24). Five-year DSS for CRC patients with low expression was 60.3% (95% CI 56.6-64.0%), for those with moderate expression 52.8% (95% CI 41.0-64.6%),and for those with high expression 8.3% (95% CI −7.4-24.0%) (Figure 4).

Cox regression univariable analysis confirmed these results. In multivariable survival analyses adjusted for age, gender, Dukes classification, and differentiation grade, membranous PODXL expression by the pAb remained statistically significant. Also the combined high expression of PODXL using mAb and pAb remained statistically significant in multivariable analysis (Table 3).

**Table 3:** Cox uni-and multivariable analysis of relative risk of death from colorectal cancer within 5 years by PODXL expression

**Polyclonal antibody**				**Monoclonal antibody**				**Combined**			
**PODXL expression**	**HR (95% CI)**	**P-value**	**N (events)**	**PODXL expression**	**HR (95% CI)**	**P-value**	**N (events)**	**PODXL expression**	**HR (95% CI)**	**P-value**	**N (events)**
	**Univariable**				**Univariable**				**Univariable**		
Non-membranous	1.00		717 (266)	Low	1.00		723 (266)	Low	1.00		714 (261)
Membranous	1.90 (1.32-2.75)	0.001	63 (32)	High	2.00 (1.31-3.06)	0.001	44 (23)	Moderate	1.38 (0.96-1.97)	0.084	79 (33)
								High	6.00 (3.27-13.0)	< 0.001	14 (11)
	**Multivariable**				**Multivariable**				**Multivariable**		
Non-membranous	1.00		712 (266)	Low	1.00		719 (266)	Low	1.00		709 (261)
Membranous	1.64 (1.11-2.43)	0.012	63 (32)	High	1.82 (1.15-2.86)	0.01	44 (23)	Moderate	1.63 (1.11-2.39)	0.012	79 (33)
								High	2.14 (1.12-4.07)	0.021	14 (11)

*Abbreviations*: *CI* confidence interval, *HR* Hazard ratio. Multivariable analysis included adjustment for gender, age (>/≤65 years), Dukes class, differentiation grade (G1/2 vs G3/4). Results of the monoclonal antibody alone have been reported previously [21].

## Discussion

Here we show that membranous PODXL expression by the pAb is an independent marker of poor prognosis in CRC. We also show that the case-by-case expression of PODXL by mAb HES9 and pAb HPA 2110 do not correlate, even though their prognostic profile and association with clinicopathological parameters (except for tumour side) is similar. Combination of the results of both antibodies enlarges the group of patients with poor prognosis compared to the use of a single antibody and revealed a group with an even worse prognosis.

As an anti-adhesive molecule, aberrant PODXL expression has been suggested to support the disruption of cell-to-cell and cell-to-extracellular matrix adhesions, thus promoting tumour dissemination [15]. Its ectopic expression has been shown to correlate with increased invasion in breast and prostate cancer [25]. Membranous PODXL expression by the polyclonal antibody HPA 2210 correlates with poor differentiation, advanced disease stage, and poor survival in CRC [17,20]. Our results confirm these findings. The results are similar, except for tumour side, to those obtained by monoclonal antibody HES9 in the same patient cohort.

Even though the two antibodies were known to recognise different epitopes within the extracellular portion of the PODXL molecule, it was surprising that their expression patterns varied and that case-by-case expressions were not uniform. It is possible that these two antibodies describe a slightly different biological phase of PODXL in CRC. This hypothesis is supported by the finding that patients with concomitant high cytoplasmic PODXL expression by the mAb and membranous expression by the pAb had an even worse DSS compared to those with only membranous or only high cytoplasmic PODXL expression. Over 70% of patients with concomitant positivity had metastatic disease and the five-year DSS was as low as 8.3%, with one-year DSS of 25.0%. The high proportion of metastasised cancers in this group supports the role of PODXL overexpression in tumour cell dissemination leading to metastases to distant organs. We hypothesise that the polyclonal antibody recognises an active form of PODXL at the cell membrane, whereas the monoclonal antibody with its smaller target epitope is able to recognise overexpression of cytoplasmic PODXL, that either has a function in the cytoplasm, or is moving towards the cell membrane. Another possibility is that these antibodies recognise different variants of PODXL. Since expression by these two antibodies seems to describe different of PODXL function, their combination provides synergy in predicting outcome.

There was no clear difference between the two antibodies as prognostic markers, as their hazard ratios for the 5-year risk of death were almost the same, with both remaining independent prognostic factors in multivariable analysis. The staining differences were clearer by the mAb and were easier to score than by the pAb. The pAb recognised a slightly larger group of patients with poor prognosis than the mAb. By the pAb there was no difference in expression between right- or left sided tumours, which we saw by the mAb [21]. This may be due to a different cytoplasmic activity of PODXL in left- compared to right-sided tumours. Verification of our finding by the mAb requires validation in other CRC patient cohorts. Moreover, further experiments on PODXL’s behaviour in CRC are needed to define the reason for this difference in expression between the antibodies and its biological background.

The proportion of membranous positivity by the pAb and the staining was obviously similar, to that of other studies on CRC and also its association to DSS and to clinicopathological parameters is corresponding [17,20]. Although the case-by-case expression differs between pAb and mAb there was no difference in association between PODXL and DSS nor with clinicopathological parameters. This supports the theory that also cytoplasmic overexpression of PODXL and not only membranous expression is a marker of poor prognosis.

The strength of this study is a well-characterised and a large patient cohort, with a long follow-up time. The TMA technique allows analysis of such large patient cohorts. Previously, the staining of PODXL in TMA-sections versus whole tissue was shown to be uniform [17], which eliminates the issue of investigating only a small proportion of the tumours by the TMA technique. Unfortunately, during the production and staining of the TMA:s 7.4% of tissue samples were lost due to technical reasons.

The different expression patterns of the two antibodies offer a possibility for their combined use. A simple combination of the expressions created two new groups; one with low cytoplasmic/non-membranous and other with high cytoplasmic and/or membranous expression. This defined a larger number of patients with poor prognosis, than either antibody alone.

When combining the expression patterns into three new classes, we were able to identify a small group of patients with a grim prognosis. The size of this group was small, and thus this phenomenon is more of biological interest than of clinical value.

## Conclusion

PODXL is an independent marker of poor prognosis in colorectal cancer. Not only membranous expression of PODXL by a polyclonal antibody (HPA 2110) shown here and in previous reports, but also high cytoplasmic expression of PODXL by monoclonal antibody (HES9) defines a group with poor prognosis. Combination of two antibodies defines a larger number of patients with poor prognosis and also a small group of patients with an even worse prognosis. This provides clues for the function of PODXL in CRC. The different expression patterns of the two antibodies suggests that they either recognise different variants of PODXL in colorectal cancer cells or that the antibodies catch PODXL at different stage on its way from cytoplasm to cellular membrane.

## Competing interests

The authors declare that they have no competing interests.

## Authors’ contributions

TK performed the statistical analyses, participated in data collection, participated in antibody scoring, and drafted the manuscript. CF and ON provided the antibody HES9 participated in study planning. JH was responsible for scoring of antibody staining and helped to draft the manuscript. HM was responsible for statistical analyses. CB participated in data collection and figure design. CH planned the study, was responsible for the immunohistochemical methods, and helped to draft the manuscript. All authors read and approved the final manuscript.

## Pre-publication history

The pre-publication history for this paper can be accessed here:

http://www.biomedcentral.com/1471-2407/14/494/prepub

## References

[B1] Siegel R Naishadham D Jemal A Cancer statistics, 2012 CA Cancer J Clin 2012 62 10 29 22237781 10.3322/caac.20138

[B2] O’Connor ESE Greenblatt DYD LoConte NKN Gangnon RER Liou J-IJ Heise CPC Smith MAM Adjuvant chemotherapy for stage II colon cancer with poor prognostic features J Clin Oncol 2011 29 3381 3388 21788561 10.1200/JCO.2010.34.3426 PMC3164243

[B3] Kerjaschki D Noronha-Blob L Sacktor B Farquhar MG Microdomains of distinctive glycoprotein composition in the kidney proximal tubule brush border The Journal of cell biology 1984 98 4 1505 1513 6371023 10.1083/jcb.98.4.1505 PMC2113241

[B4] Horvat RR Hovorka AA Dekan GG Poczewski HH Kerjaschki DD Endothelial cell membranes contain podocalyxin–the major sialoprotein of visceral glomerular epithelial cells J Cell Biol 1986 102 484 491 3511072 10.1083/jcb.102.2.484 PMC2114082

[B5] Somasiri A Nielsen JS Makretsov N McCoy ML Prentice L Gilks CB Chia SK Gelmon KA Kershaw DB Huntsman DG McNagny KM Roskelley CD Overexpression of the anti-adhesin podocalyxin is an independent predictor of breast cancer progression Cancer Res 2004 64 5068 5073 15289306 10.1158/0008-5472.CAN-04-0240

[B6] Doyonnas RR Nielsen JSJ Chelliah SS Drew EE Hara TT Miyajima AA McNagny KMK Podocalyxin is a CD34-related marker of murine hematopoietic stem cells and embryonic erythroid cells Blood 2005 105 4170 4178 15701716 10.1182/blood-2004-10-4077

[B7] Kershaw DBD Beck SGS Wharram BLB Wiggins JEJ Goyal MM Thomas PEP Wiggins RCR Molecular cloning and characterization of human podocalyxin-like protein. Orthologous relationship to rabbit PCLP1 and rat podocalyxin J Biol Chem 1997 272 15708 15714 9188463 10.1074/jbc.272.25.15708

[B8] Richards MM Tan S-PS Tan J-HJ Chan W-KW Bongso AA The transcriptome profile of human embryonic stem cells as defined by SAGE Stem Cells 2003 22 51 64 14688391 10.1634/stemcells.22-1-51

[B9] Nielsen JS McNagny KM The role of podocalyxin in health and disease J Am Soc Nephrol 2009 20 1669 1676 19578008 10.1681/ASN.2008070782

[B10] Dallas MR Chen S-H Streppel MM Sharma S Maitra A Konstantopoulos K Sialofucosylated podocalyxin is a functional E- and L-selectin ligand expressed by metastatic pancreatic cancer cells Am J Physiol Cell Physiol 2012 303 C616 C624 22814396 10.1152/ajpcell.00149.2012 PMC3468350

[B11] Konstantopoulos K Thomas SN Cancer cells in transit: the vascular interactions of tumor cells Annu Rev Biomed Eng 2009 11 177 202 19413512 10.1146/annurev-bioeng-061008-124949

[B12] Thomas SN Schnaar RL Konstantopoulos K Podocalyxin-like protein is an E-/L-selectin ligand on colon carcinoma cells: comparative biochemical properties of selectin ligands in host and tumor cells Am J Physiol Cell Physiol 2009 296 C505 C513 19118161 10.1152/ajpcell.00472.2008 PMC2660269

[B13] Meng X Ezzati P Wilkins JA Requirement of podocalyxin in TGF-beta induced epithelial mesenchymal transition PLoS One 2011 6 e18715 21533279 10.1371/journal.pone.0018715 PMC3075272

[B14] Casey GG Neville PJP Liu XX Plummer SJS Cicek MSM Krumroy LML Curran APA McGreevy MRM Catalona WJW Klein EAE Witte JSJ Podocalyxin variants and risk of prostate cancer and tumor aggressiveness Hum Mol Genet 2006 15 735 741 16434482 10.1093/hmg/ddi487

[B15] Nielsen JS Graves ML Chelliah S Vogl AW Roskelley CD McNagny KM The CD34-related molecule podocalyxin is a potent inducer of microvillus formation PLoS One 2007 2 e237 17311105 10.1371/journal.pone.0000237 PMC1796660

[B16] Hsu Y-HY Lin W-LW Hou Y-TY PY-SY Shun C-TC Chen C-LC WY-YY Chen J-YJ Chen T-HT Jou T-ST Podocalyxin EBP50 ezrin molecular complex enhances the metastatic potential of renal cell carcinoma through recruiting Rac1 guanine nucleotide exchange factor ARHGEF7 Am J Pathol 2010 176 12 12 10.2353/ajpath.2010.090539 PMC2877864 20395446

[B17] Larsson A Johansson ME Wangefjord S Gaber A Nodin B Kucharzewska P Welinder C Belting M Eberhard J Johnsson A Uhlén M Jirström K Overexpression of podocalyxin-like protein is an independent factor of poor prognosis in colorectal cancer Br J Cancer 2011 105 666 672 21829192 10.1038/bjc.2011.295 PMC3188928

[B18] Boman K Larsson AH Segersten U Kuteeva E Johannesson H Nodin B Eberhard J Uhlén M Malmström P-U Jirström K Membranous expression of podocalyxin-like protein is an independent factor of poor prognosis in urothelial bladder cancer Br J Cancer 2013 108 11 2321 2328 23652315 10.1038/bjc.2013.215 PMC3681027

[B19] Schopperle WM Kershaw DB DeWolf WC Human embryonal carcinoma tumor antigen, Gp200/GCTM-2, is podocalyxin Biochem Biophys Res Commun 2003 300 285 290 12504081 10.1016/s0006-291x(02)02844-9

[B20] Larsson A Fridberg M Gaber A Nodin B Levéen P Jönsson G Uhlén M Birgisson H Jirström K Validation of podocalyxin-like protein as a biomarker of poor prognosis in colorectal cancer BMC Cancer 2012 12 282 282 22769594 10.1186/1471-2407-12-282 PMC3492217

[B21] Kaprio T Fermér C Hagström J Mustonen H Böckelman C Nilsson O Haglund C Podocalyxin is a marker of poor prognosis in colorectal cancer BMC Cancer 2014 14 493 25004863 10.1186/1471-2407-14-493 PMC4226963

[B22] Kononen J Bubendorf L Kallionimeni A Bärlund M Schraml P Leighton S Torhorst J Mihatsch MJ Sauter G Kallionimeni O-P Tissue microarrays for high-throughput molecular profiling of tumor specimens Nat Med 1998 4 844 847 9662379 10.1038/nm0798-844

[B23] Uhlens M Bjorling E Agaton C Szigyarto CA Amini B Andersen E Andersson AC Angelidou P Asplund A Asplund C Berglund L Bergstrom K Brumer H Cerjan D Ekstrom M Elobeid A Eriksson C Fagerberg L Falk R Fall J Forsberg M Bjorklund MG Gumbel K Halimi A Hallin I Hamsten C Hansson M Hedhammar M Hercules G Kampf C A human protein atlas for normal and cancer tissues based on antibody proteomics Mol Cell Proteomics 2005 4 1920 1932 16127175 10.1074/mcp.M500279-MCP200

[B24] Pontèn F Jirström K Uhlén M The human protein atlas-a tool for pathology J Pathol 2008 216 387 393 18853439 10.1002/path.2440

[B25] Sizemore SS Cicek MM Sizemore NN Ng KPK Casey GG Podocalyxin increases the aggressive phenotype of breast and prostate cancer cells in vitro through its interaction with ezrin Cancer Res 2007 67 6183 6191 17616675 10.1158/0008-5472.CAN-06-3575

